# Prevalence of Systemic Diseases in Patients Attending a Dental Clinic. A cross-sectional study

**DOI:** 10.4317/jced.63930

**Published:** 2026-05-29

**Authors:** Carmen López-Carriches, Hujjatullah Ghaffari, Ricardo Taheri, Jorge Cortés-Bretón-Brinkmann, Fabián Pérez-González, Isabel Leco-Berrocal, Cristina Meniz-García

**Affiliations:** 1 Associate Professor. Department of Dental Clinical Specialties. School of Dentistry. Universidad Complutense de Madrid, Spain; 2 Doctor of Dental Surgery (DDS). Collaborator. School of Dentistry. Universidad Complutense de Madrid, Spain; 3 Assistant Professor. Department of Dental Clinical Specialties. School of Dentistry. Universidad Complutense de Madrid, Spain

## Abstract

**Background:**

It is crucial to identify the diseases and chronic conditions affecting patients attending a dental clinic, particularly in the field of oral surgery. Dentists must be provided with this information in order to reduce the risk of complications and medical emergencies when treating their patients.

**Materials and Methods:**

An observational, retrospective, cross-sectional study was conducted on 862 patients attending a dental clinic over a 6-month period. Data obtained from medical questionnaires were analyzed using descriptive statistics to determine patients' age, sex, and reported systemic diseases and conditions. Microsoft Excel and Python were used for data analysis.

**Results:**

Allergies, cardiovascular disease, and metabolic disorders were the most commonly reported conditions among patients attending the dental clinic, making them the conditions most frequently encountered in this patient population.

**Conclusions:**

A thorough medical history should be obtained for all dental patients to ensure safe dental care. Identification and consideration of chronic systemic diseases during treatment planning are essential to minimize the risk of intraoperative and postoperative complications, particularly in oral surgery.

## Introduction

As dentists, we treat patients with chronic diseases on a daily basis. Therefore, before providing care, the patient should be assessed through a careful medical history and an evaluation of the patient's ability to tolerate dental treatment. Systemic conditions may influence treatment planning and increase the risk of complications during dental and oral surgical procedures. The prevalence of systemic diseases among dental patients has been reported in previous studies; however, these data may vary depending on geographic location, population characteristics, and the type of dental care setting. Moreover, the increasing prevalence of chronic diseases in the general population highlights the need for updated epidemiological information from specific clinical environments. Identifying the most common systemic conditions encountered in dental practice may assist clinicians in anticipating potential complications, adjusting pharmacological prescriptions, and improving patient safety during dental and oral surgical procedures. The primary objective of this study was to determine the prevalence of systemic diseases and chronic conditions among patients attending a dental clinic. We hypothesized that a substantial proportion of dental patients would present at least one chronic systemic condition.

## Materials and Methods

A retrospective, cross-sectional study was conducted on 862 patients who attended a private dental clinic over a 6-month period, during the year 2025. Medical information was collected using a standardized medical history questionnaire completed by patients before receiving dental care. The questionnaire follows the anamnesis format recommended by the Official College of Dentists of Madrid and is routinely used in dental practice to systematically document patients' medical history. The variables collected included patient age, sex, and the presence of self-reported systemic diseases and medical conditions. The medical conditions recorded included cardiovascular diseases, metabolic disorders, respiratory diseases, allergies, endocrine disorders, neurological conditions, and other relevant health-related factors such as tobacco and alcohol consumption. Patients were asked about previously diagnosed systemic conditions and current medical treatments, and this information was recorded in the dental clinical chart. Patients were allowed to report more than one systemic disease; therefore, each condition was recorded as an independent variable and a single patient could be included in more than one disease category. Consecutive sampling was used in order to minimize selection bias and reflect routine clinical practice. Only patients with complete medical questionnaires in their clinical records were included in the analysis. Patients with incomplete or missing medical history information were excluded. All patient data were anonymized prior to analysis to ensure confidentiality and compliance with ethical standards. Data were compiled into spreadsheets and analyzed using descriptive statistical methods. Microsoft Excel and Python software were used for data management and statistical analysis. Descriptive statistics were applied to assess age distribution, sex, and the prevalence of the most common chronic systemic diseases in the study population. A proxy variable for depression/anxiety was generated based on the use of antidepressant and/or anxiolytic medications. The prevalence of each disease/condition was calculated as the proportion of affected patients in the sample. Estimates are reported with 95% confidence intervals (95% CI) calculated using the Wilson method to provide an estimate of the uncertainty around the observed prevalence. Stratified analyses were performed by age group (65 years) and sex. Associations were assessed using the chi-square test or Fisher's exact test when appropriate.

## Results

**Figure 1:**
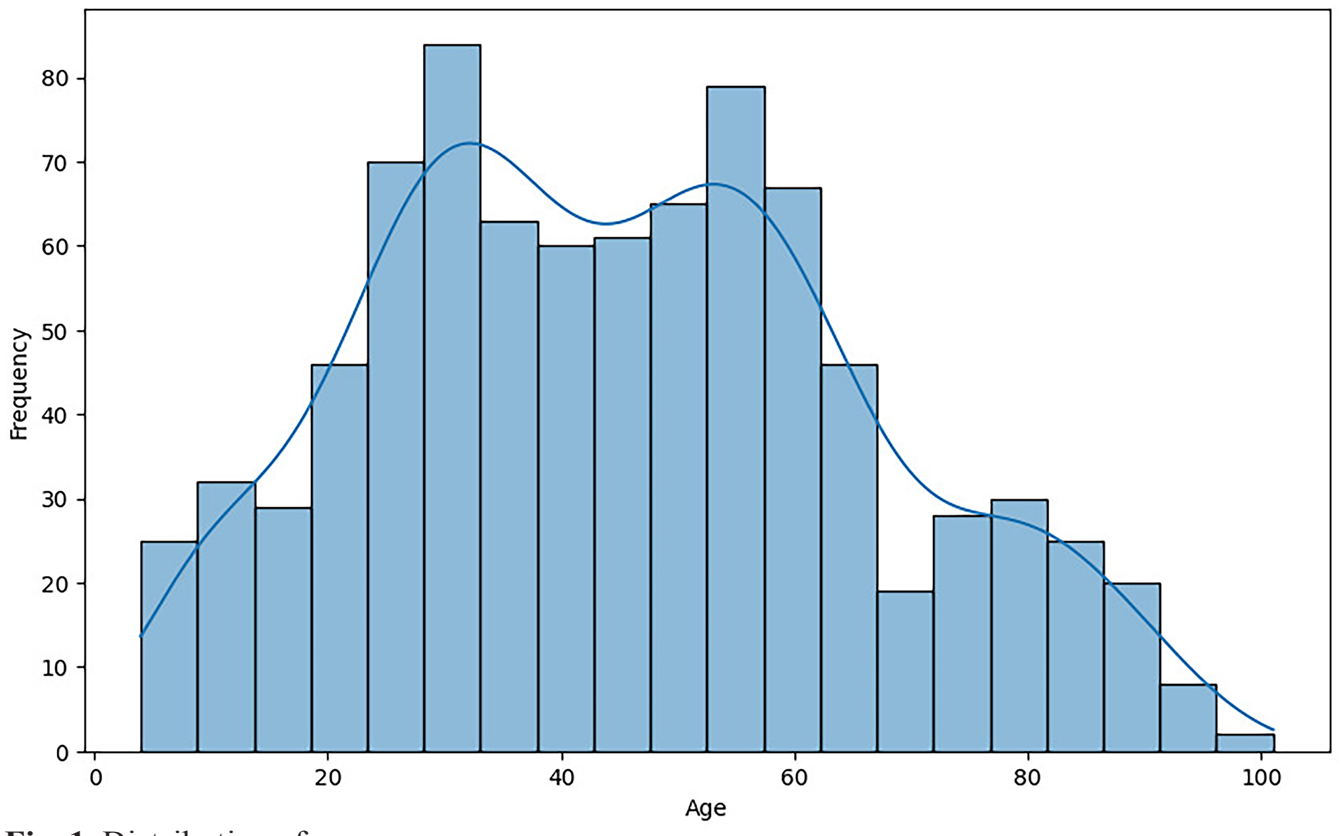
Distribution of age.

- Demographic characteristics The mean age was 45.41 years (range: 4-101 years) (Fig. 1).

1

**Figure 2:**
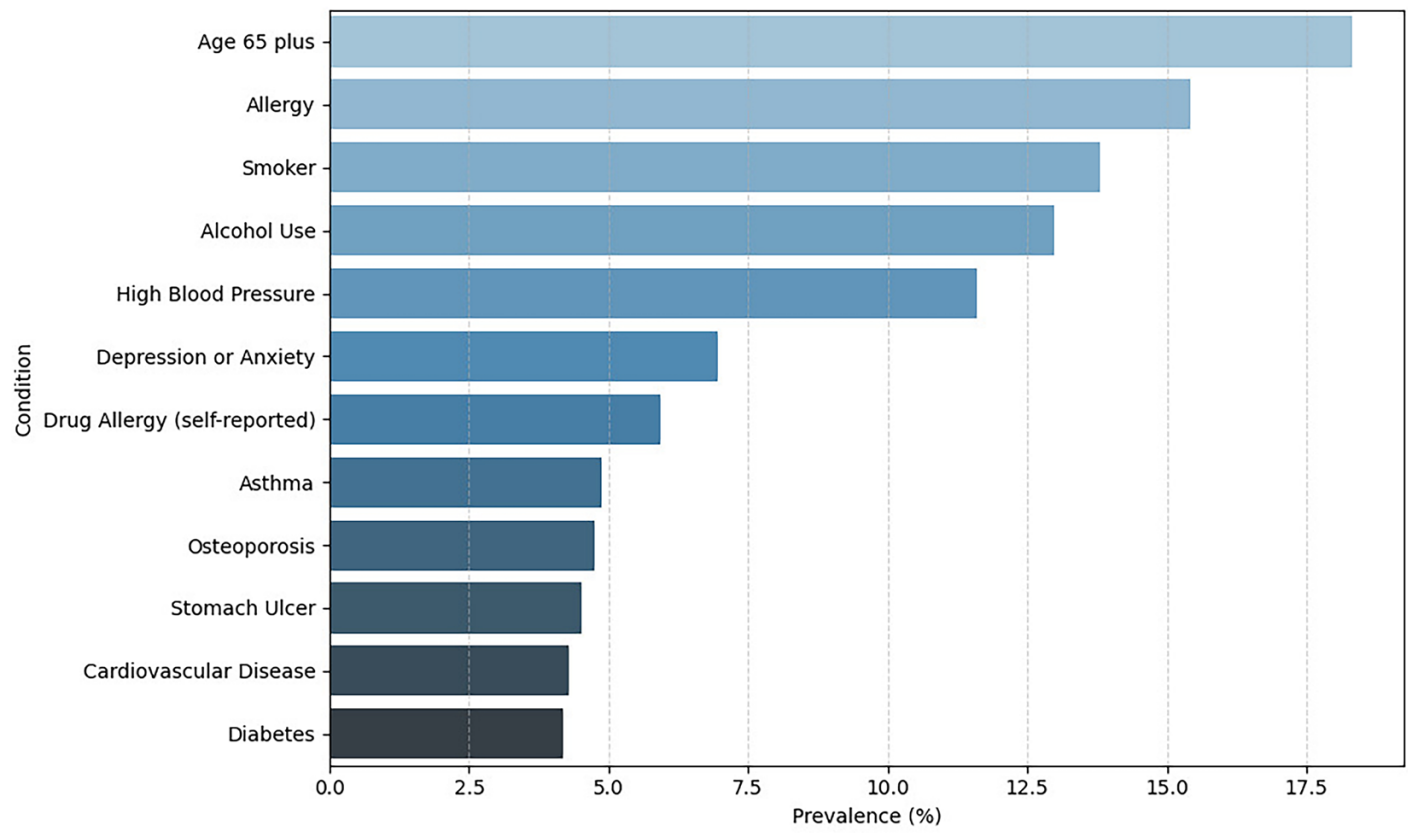
Most frequent diseases/conditions.

Although most patients were adults, a substantial proportion of the sample included older individuals, reflecting a heterogeneous population in terms of age. Women constituted a slightly higher proportion of the sample (57.1%) compared with men (42.9%). - Prevalence of systemic diseases and health-related factors Regarding medical background, a broad spectrum of systemic conditions and health-related behaviours was identified (Table 1, Fig. 2).

Table 1

2

- Stratified Analysis Stratified analysis revealed significant differences according to age. Cardiovascular disease and hypertension were markedly more prevalent among patients aged 65 years (both p &lt; 0.001). In addition, older patients showed significantly higher prevalence of diabetes, osteoporosis, hypothyroidism, and neurodegenerative conditions such as Parkinson's disease (all p &lt; 0.05). In contrast, lifestyle-related factors such as tobacco and alcohol use were significantly more frequent among younger patients (both p &lt; 0.01). No statistically significant differences by age were observed for several less prevalent conditions. Regarding sex, significant differences were observed for hypothyroidism, hypertension, osteoporosis, and drug use (p &lt; 0.05), whereas no differences were found for the remaining conditions. The variable for depression/anxiety showed a significantly higher prevalence among patients aged 65 years compared with younger individuals (12.0% vs. 5.8%, p = 0.010). Additionally, this condition was more frequently observed in women than in men (8.5% vs. 4.9%, p = 0.050). A total of 58.5% of patients presented at least one systemic condition, while 27.8% showed multimorbidity (2 conditions). Multimorbidity was significantly more prevalent among patients aged 65 years compared with younger individuals (46.8% vs. 23.6%, p &lt; 0.001). A higher prevalence of multimorbidity was also observed in women compared to men (30.7% vs. 24.1%). These findings indicate that age plays a major role in the distribution of systemic diseases among dental patients, whereas sex-related differences appear to be more condition-specific. Allergies represented one of the most commonly reported conditions, with 15.43% of patients reporting non-drug-related allergies and 5.92% reporting drug allergies. Cardiovascular diseases were among the most prevalent medical conditions observed in the study population. Arterial hypertension was reported by 11.6% of patients, while other cardiac conditions were reported by 4.29%. Metabolic disorders were also common, with diabetes mellitus affecting 4.18% of patients. Respiratory diseases were also present in the study population, with asthma reported by 4.80% of patients. In addition, mental health conditions were relatively frequent, as 8.80% of patients reported a history of depression or anxiety. Several health-related behaviors associated with systemic and oral health risks were also identified. Tobacco use was reported by 13.81% of patients, while alcohol consumption was reported by 12.99%. Less frequently reported conditions included kidney disease (2.09%), epilepsy (1.97%), cancer (1.39%), dermatological diseases (0.50%), and neurodegenerative disorders (0.30%). Only one patient (0.12%) reported a transmissible infectious disease such as HIV.

## Discussion

**Figure 3:**
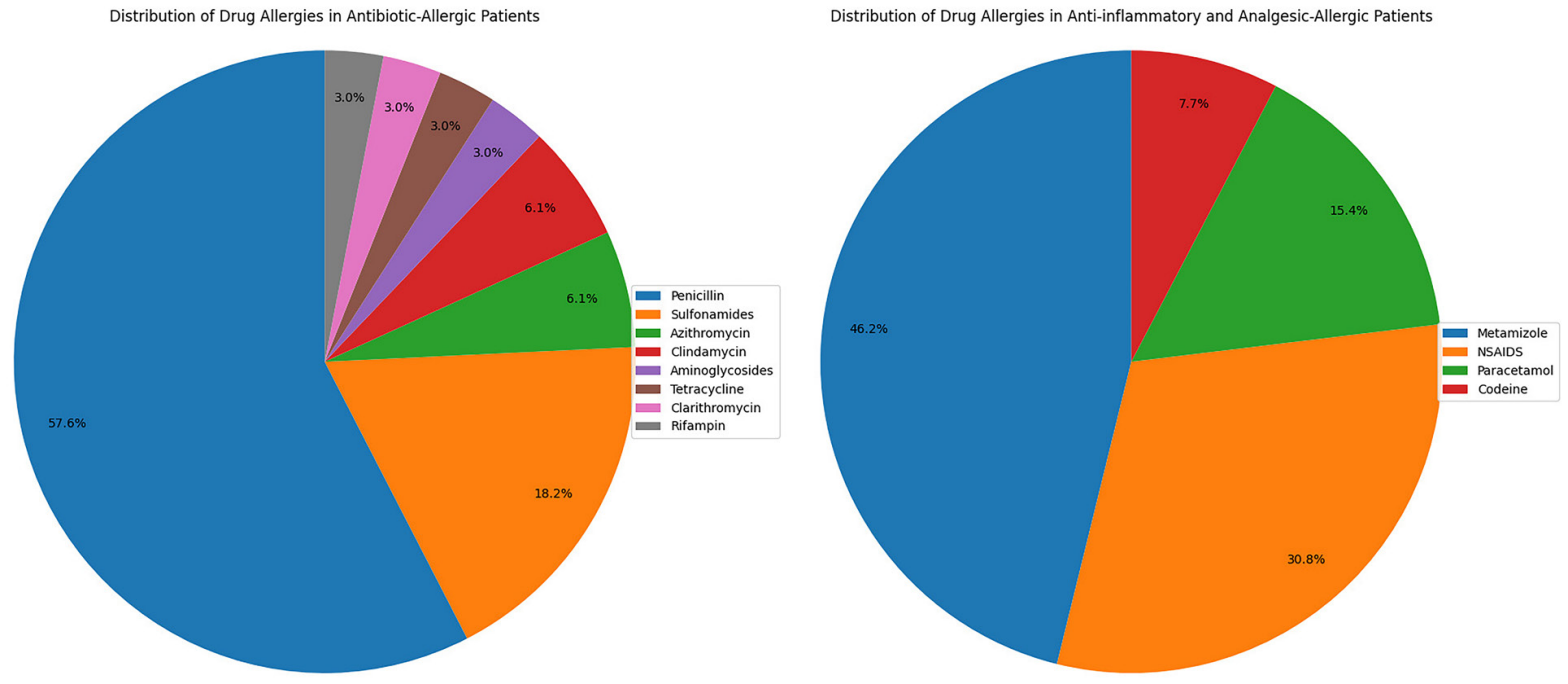
Distribution of allergies.

A considerable proportion of the study population consisted of older adults. Increasingly, dentists are treating elderly, polymedicated patients. The global growth of the older population is associated with a higher prevalence of agerelated health problems and substantial public healthcare costs. From a dental perspective, the management of these patients is also more complex. The high prevalence of multimorbidity observed in this study further highlights the clinical complexity of patients attending dental practice. More than one quarter of patients presented two or more systemic conditions, with a markedly higher prevalence among older individuals. This finding reflects the cumulative nature of chronic diseases with aging and has important implications for dental care, including increased polypharmacy and a higher risk of medical complications during treatment. In our sample, 15.43% of patients reported non-drug-related allergies. Although these allergies generally do not interfere with routine dental practice, special considerations are required when they are associated with respiratory conditions such as asthma. Additionally, 5.92% of patients reported drug allergies, most commonly to antibiotics (64.71%). The most frequent antibiotic allergy was to penicillin (19 patients; 2.20%), followed by sulfonamides (6 patients; 0.70%). Regarding analgesics and antiinflammatory agents, allergy was reported to metamizole/dipyrone (6 patients; 0.70%), NSAIDs (4 patients; 0.46%), paracetamol/acetaminophen (2 patients; 0.23%), and codeine (1 patient; 0.12%) (Fig. 3).

3

Dentists frequently prescribe antibiotics and anti-inflammatory medications as part of routine care. Appropriate alternative pharmacological options should be considered for patients with known drug allergies (1). A substantial proportion of patients reported smoking. As oral healthcare professionals, we should actively encourage smoking cessation, given its well-established association with oral cancer and other adverse oral outcomes. The same applies to alcohol consumption, which is also linked to increased oral disease risk and can further amplify the harmful effects of tobacco when both are present. Cardiovascular disease represents one of the most frequent and clinically relevant medical conditions in dental patients. In our study, cardiovascular disease affected men more than women (54.05% vs. 45.95%). However, cardiovascular problems have been reported to be under-diagnosed in women. Cardiovascular diseases account for more than 25% of deaths worldwide. The most common cardiovascular condition is ischaemic heart disease (IHD). Older adults and specially men are the most affected populations (2). The American Heart Association has promoted lifelong cardiovascular health through the concept of "Life's Essential 8", which includes diet, physical activity, nicotine exposure, sleep health, body mass index, blood lipids, blood glucose, and blood pressure (3). Given the large number of patients with cardiovascular conditions treated in dental practice, dentists are well positioned to support prevention efforts and promote healthier behaviors in all their susceptible patients. Many patients with cardiovascular disease are receiving anticoagulant or antiplatelet therapy, which has important implications for dental and surgical procedures. In addition, antibiotic prophylaxis should be considered when indicated, to prevent infective endocarditis. Having identified these high-risk patients, recommended prophylaxis is amoxicillin (2 g orally). For patients allergic to penicillin, recommended alternatives include cephalexin (2 g orally), azithromycin (500 mg orally), clarithromycin (500 mg orally), or doxycycline (100 mg orally). These pharmacological treatments are administered as a single dose 30-60 minutes before the procedure. Clindamycin is discouraged in patients allergic to betalactams due to the higher risk of Clostridium difficile infection; macrolides or doxycycline are preferred instead (4). Equally important, patients with myocardial ischaemia (angina pectoris or a history of acute myocardial infarction) should not be subjected to stress. Appointments should be short, after providing thorough explanations and addressing questions. When needed, a benzodiazepine may be prescribed. In the event of an emergency, the medication of choice is sublingual nitroglycerin, which should be an integral component of the dental emergency kit. Dentists must also be trained to recognize symptoms such as chest pain, etc (5). A high proportion of our patients were identified with arterial hypertension, which can contribute to cardiac damage with subsequent risk of cardiovascular events, as well as renal damage leading to kidney failure. The European Society of Cardiology/European Society of Hypertension (ESC/ESH) defines hypertension as systolic BP 140 mmHg and/or diastolic BP 90 mmHg, whereas the American College of Cardiology/American Heart Association (ACC/AHA) defines hypertension as systolic BP 130 mmHg or diastolic BP 80 mmHg (6). Potential interactions should be considered with NSAIDs, which may reduce the effectiveness of antihypertensive drugs. Short morning appointments are preferable; diazepam (5-10 mg) may be used when indicated. Also, sudden changes in chair position should be avoided to reduce the risk of orthostatic hypotension. Pain control is essential; local anaesthetic with a vasoconstrictor is often preferable; the use of up to two cartridges of lidocaine 1:100.000 (0.018 mg of adrenaline) is recommended, or alternatively up to four cartridges of articaine 1:200.000, which contains 0.009 mg of adrenaline per cartridge. Also, during surgical procedures, hypertensive patients may bleed more; extensive interventions should be avoided (5). In our study, 4.87% of patients reported asthma in their medical history. These patients had a mean age of 65.5 years. Seventysix patients (8.80%) were identified based on antidepressant and/or anxiolytic medication use, suggesting the presence of depression or anxiety. Because of their medications, these patients may present with xerostomia and also increased risk of caries, candidiasis and other infections (7). Regarding dermatological problems only five patients reported taking isotretinoin. Oral retinoids used for acne may impair wound healing. They may also reduce salivary flow, thereby increasing the risk of caries (8). A relevant proportion of patients reported osteoporosis. This is clinically important because bisphosphonate therapy, particularly systemic administration, may be associated with medicationrelated osteonecrosis of the jaw (MRONJ) after tooth extractions and implant placement. Therefore, these patients should attend regular dental followups to control caries, periodontal disease, and other oral and dental conditions (9). Risk may be low, moderate, or high depending on the bisphosphonate type, duration of therapy, and route of administration. Medical consultation may be required to determine whether temporary discontinuation of therapy is appropriate. For intravenous bisphosphonate, therapy is generally not suspended. If extraction is necessary, the most suitable timing is to postpone the procedure as long as possible from the last dose received. Preventive measures include chlorhexidine mouth rinses, antibiotic prophylaxis when indicated, atraumatic extraction, primary closure with suturing, and postoperative reviews. Whether discontinuation reduces the risk of MRONJ remains debated. If oral therapy is suspended, risk appears lower when the interruption exceeds 90 days and minimal when the drug interruption is longer than one year. Risk is lower with ibandronate and higher with zoledronate (10). Also, bisphosphonates may interact with NSAIDs, as both can affect the gastrointestinal mucosa. Therefore, gastrointestinal protection should be considered (11). Diabetes mellitus was the most frequent metabolic disease among our patients. More than 9% of the world's adult population has diabetes, and this figure continues to rise. Therefore, patients should be counselled about healthy lifestyle habits; dietary modification and physical exercise represent non-pharmacological management options for these patients, when feasible (12). Epinephrine increases glycogenolysis and may cause hyperglycemia; this should be considered in patients with type 2 diabetes (13). Dentists can support the diagnosis of diabetes. These patients may present with periodontal disease, caries, fungal infections, and increased susceptibility to infection. A fasting blood glucose test may be ordered, or the patient may be referred to a physician if diabetes is suspected. It is important to determine whether the patient's diabetes is well controlled (14). Hypothyroidism was detected relatively frequently, although lower than the 4-10% incidence has been reported in dental literature (15). Patients may present with fatigue, increased sensitivity to cold, constipation, dry skin, weight gain, facial swelling, hoarseness, coarse hair, and rough skin. They may metabolize drugs poorly and may have associated cardiac problems. Sedatives should be avoided (16). Some studies report a positive association between periodontitis and hypothyroidism (17). The converse condition, hyperthyroidism, was reported by 1.74% of patients. Symptoms may include palpitations, fatigue, weight loss, heat intolerance, anxiety, and tremor. Renal function must be considered because local anaesthetics used in dental procedures are eliminated via the kidneys. The lowest effective dose should be administered in these patients. Articaine is often regarded as the gold-standard option for local anesthesia in this setting (18). Nephrotoxic drugs should be avoided, including aminoglycosides (gentamicin and streptomycin), tetracyclines, and cephalosporins (19 , 20). Moreover, the relationship between hyperuricaemia and periodontitis is an area of ongoing research (21). Epilepsy was not frequent in our sample; however, seizures may still occur in the dental office. Diazepam for emergency use should always be available in the emergency kit. In addition, the dental team should be prepared to protect the patient during an episode by stopping treatment, removing instruments from the mouth, positioning the patient safely, and maintaining a clear airway to reduce the risk of injury or aspiration (22). Only one patient reported HIV infection. In patients with HIV or other bloodborne infections, safetyengineered needles that do not require recapping should be considered. Human immunodeficiency virus (HIV) infection affects millions of people worldwide (23). It is also possible that some patients may not disclose this condition in their medical history. In our study, 1.39% of patients were undergoing cancer treatment. It is important to monitor these patients and apply preventive protocols to avoid and manage oral complications (24). Some limitations of this study should be acknowledged. The data were obtained from self-reported medical questionnaires, which may be subject to reporting bias or incomplete disclosure of certain conditions. In addition, the study was conducted in a single private dental clinic, which may limit the generalizability of the findings. Despite these limitations, the study provides useful insight into the systemic health conditions commonly encountered in routine dental practice and highlights the importance of thorough medical history assessment before dental treatment.

## Conclusions

Dental clinics provide care to patients with a wide range of systemic diseases; therefore, maintaining an up-to-date medical history is essential to prevent treatment-related complications, implement appropriate precautions, including adjustments to anesthesia, medication, and surgical procedures. Ultimately, a thorough and comprehensive medical evaluation enhances patient safety, improves clinical outcomes, and reduces the risk of medical emergencies during dental treatment.

## Figures and Tables

**Table 1:** Proportion of patients with each disease/condition.

Disease / Condition	% of Patients	CI 95%
Older than 65 years	17.52%	[15.13 – 20.20]
Non‑drug allergies	15.43%	[13.17 – 17.99]
Tobacco use	13.81%	[11.66 – 16.27]
Alcohol consumption	12.99%	[10.91 – 15.4]
Hypertension	11.6%	[9.63 – 13.91]
Depression or anxiety	8.80%	[7.10 – 10.90]
Drug allergies	5.92%	[4.53 – 7.70]
Asthma	4.80%	[3.62 – 6.52]
Osteoporosis	4.76%	[3.53 – 6.39]
Stomach ulcer / gastritis	4.52%	[3.33 – 6.13]
Other cardiac conditions	4.29%	[3.13 – 5.86]
Diabetes mellitus	4.18%	[3.03 – 5.73]
Hypothyroidism	2.44%	[1.60 – 3.70]
Kidney disease	2.09%	[1.32 – 3.28]
Epilepsy	1.97%	[1.23 – 3.14]
Hyperthyroidism	1.74%	[1.06 – 2.85]
Cancer	1.39%	[0.80 – 2.42]
Dermatological diseases	0.50%	[0.19 – 1.29]
Neurodegenerative diseases	0.30%	[0.10 – 1.02]
Transmissible infectious diseases such as HIV	0.12%	[0.02 – 0.65]

1
